# The potential of using blood circular RNA as liquid biopsy biomarker for human diseases

**DOI:** 10.1007/s13238-020-00799-3

**Published:** 2020-11-01

**Authors:** Guoxia Wen, Tong Zhou, Wanjun Gu

**Affiliations:** 1 State Key Laboratory of Bioelectronics, School of Biological Sciences and Medical Engineering, Southeast University, Nanjing, 210096 China; 2 Department of Physiology and Cell Biology, Reno School of Medicine, University of Nevada, Reno, NV 89557 USA

**Keywords:** peripheral blood circular RNA, liquid biopsy, human diseases, translational biomarkers

## Abstract

Circular RNA (circRNA) is a novel class of single-stranded RNAs with a closed loop structure. The majority of circRNAs are formed by a back-splicing process in pre-mRNA splicing. Their expression is dynamically regulated and shows spatiotemporal patterns among cell types, tissues and developmental stages. CircRNAs have important biological functions in many physiological processes, and their aberrant expression is implicated in many human diseases. Due to their high stability, circRNAs are becoming promising biomarkers in many human diseases, such as cardiovascular diseases, autoimmune diseases and human cancers. In this review, we focus on the translational potential of using human blood circRNAs as liquid biopsy biomarkers for human diseases. We highlight their abundant expression, essential biological functions and significant correlations to human diseases in various components of peripheral blood, including whole blood, blood cells and extracellular vesicles. In addition, we summarize the current knowledge of blood circRNA biomarkers for disease diagnosis or prognosis.

## INTRODUCTION

Liquid biopsy is a biopsy that uses body liquids as the sample source to diagnose, predict the outcome of or monitor the development of human diseases (Rubis et al., 2019; Luo et al., 2020a). Compared to traditional tissue biopsy, liquid biopsy has the advantages of being noninvasive, performed in real-time and accurate (Rubis et al., 2019; Luo et al., 2020a). It has been proven to be applicable to the management of many human diseases, including cancers (Heitzer et al., 2019; Mattox et al., 2019; Rubis et al., 2019), prenatal genetic disorders (Zhang et al., 2019a), heart diseases (Zemmour et al., 2018), schizophrenia (Chen et al., 2020b), transplant rejection (Bloom et al., 2017) and infectious diseases (Burnham et al., 2018; Hong et al., 2018; Blauwkamp et al., 2019; Han et al., 2020a). To date, most liquid biopsy studies have focused on its clinical application in human cancers (reviewed in Siravegna et al., 2017; Heitzer et al., 2019; Mattox et al., 2019; Rubis et al., 2019). For example, a cell-free DNA (cfDNA)-based liquid biopsy test that determines the mutational status of the epidermal growth factor receptor (*EGFR*) gene was used to guide the response of *EGFR* tyrosine kinase inhibitors in non-small cell lung cancer (NSCLC) patients, which was approved by the FDA in clinical practice (Kwapisz, 2017). Another FDA-approved liquid biopsy test, Epi proColon, assessed the methylation status of the *Septin9* gene in whole blood, which was used to screen colorectal cancer patients from healthy controls (Lamb and Dhillon, 2017). In addition to cfDNA (Wan et al., 2017; Cescon et al., 2020), several other analytes within circulating body fluids were investigated as liquid biopsy biomarkers, such as circulating tumor cells (CTCs) (Yu et al., 2013), extracellular vesicles (EVs) (Torrano et al., 2016), cell-free RNA (cfRNA) (Zaporozhchenko et al., 2018), circulating proteins (Surinova et al., 2015), circulating metabolites (Crutchfield et al., 2016) and platelets (Joosse and Pantel, 2015; Best et al., 2017). Among them, RNA-based liquid biopsy biomarkers have gained much more attention in recent years since they have dynamic expressions and are closely related to different disease conditions (Zaporozhchenko et al., 2018; Sole et al., 2019). Circulating noncoding RNAs, especially microRNAs (miRNAs), have shown promising potential as stable blood-based biomarkers in liquid biopsy (Mitchell et al., 2008; Anfossi et al., 2018; Pardini et al., 2019).

Circular RNAs (circRNAs) are a group of endogenous noncoding RNA molecules (Chen, 2016, 2020; Li et al., 2018d). They were first found in plant viroids (Sanger et al., 1976) and eukaryotic cells (Hsu and Coca-Prados, 1979) in 1970s, and were recently observed to be functional (Hansen et al., 2013; Memczak et al., 2013). They are joined head to tail to generate a covalently closed loop structure through back-splicing (Vicens and Westhof, 2014; Li et al., 2018d). They lack a 5-prime cap and 3-prime poly-A tail, which is quite different from canonical linear RNAs (Vicens and Westhof, 2014; Li et al., 2018d). CircRNAs have been identified in almost all organisms across the eukaryotic tree of life (Wang et al., 2014). Some circRNAs are the predominant transcript isoform of their host genes expressed in specific tissues or cell types (Salzman et al., 2012, 2013; Rybak-Wolf et al., 2015). Furthermore, they are expressed in a tissue- (Zhou et al., 2018a), cell-type- (Salzman et al., 2013) and developmental stage-specific manner (Zhou et al., 2018a). Accumulating studies have revealed many regulatory roles and versatile cellular functions that circRNAs can perform (Li et al., 2018d; Chen, 2020). They can act as miRNA decoys (Hansen et al., 2013), RNA binding protein (RBP) sponges (Ashwal-Fluss et al., 2014; Huang et al., 2020a) and protein scaffolds (Li et al., 2015c, 2019a). Moreover, a small percentage of circRNAs can be translated into proteins (Legnini et al., 2017; Pamudurti et al., 2017; Heesch et al., 2019). Aberrant expression of circRNAs has been related to many human diseases, including cancers (Shang et al., 2019; Vo et al., 2019), neurodegenerative diseases, cardiovascular diseases (Aufiero et al., 2019) and immune diseases (Chen et al., 2019c; Zhou et al., 2019b). Due to their high stability (Enuka et al., 2015), abundant expression (Jeck et al., 2013) and high specificity (Zhou et al., 2018a), circRNAs are becoming promising biomarkers for human diseases (Zhang et al., 2018j).

Due to the importance of liquid biopsy biomarkers in precision medicine (Vargas and Harris, 2016) and the superior characteristics of circRNAs as disease biomarkers (Zhang et al., 2018j), recent studies have put great efforts into researching the use of circRNAs as liquid biopsy biomarkers for human diseases. Here, we review current progress that has been made on this topic. We focus on circRNAs in the peripheral blood, although circRNAs are abundantly expressed in other body fluids, such as saliva (Bahn et al., 2014; Ghods, 2018) and urine (Kölling et al., 2019; Lam and Lo, 2019; Vo et al., 2019). In this review, we first briefly introduce the biogenesis, function, and expression patterns of circRNAs and their close correlations to human diseases. Then, we emphasize the functional roles they may play in different blood components, including blood cells, serum, plasma, platelets and EVs in the blood. We summarize peripheral blood circRNA biomarkers that have been constructed for the management of human diseases. Finally, we discuss future opportunities and challenges of translating blood circRNAs into clinical practice.

## THE BIOGENESIS OF CIRCRNAS

**Figure 1:**
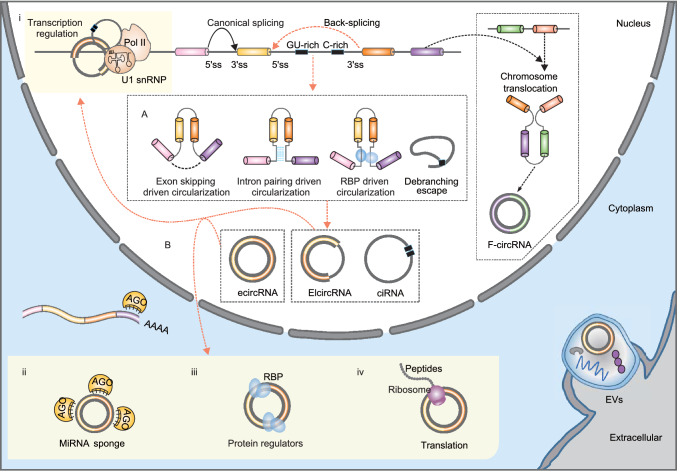
**The biogenesis and function of circRNAs.** (A) CircRNAs are formed by back-splicing of pre-mRNAs by different mechanisms, including: lariat driven circularization, intron pairing driven circulation, RBP driven circularization, and debranching escape of intron lariats. (B) CircRNAs can perform diverse biological functions. First, EIcircRNAs can interact with *RNA Pol II* and *U1 snRNP* to regulate gene transcription in the nucleus (i). Second, ecircRNAs can accumulate in the cytoplasm and act as miRNA decoys (ii), protein regulators (iii), and translation templates (iv). Third, circRNAs can be secreted into EVs by many cell types and transported to recipient cells by EVs. EV circRNAs can also act as important gene regulators

CircRNAs are derived from pre-mRNAs and formed by back-splicing, in which a downstream 5-prime splice site (ss) is covalently joined with an upstream 3-prime ss (Fig. 1) (Li et al., 2018d). There are three major types of circRNAs, exonic circRNAs (ecircRNAs), exon-intron circRNAs (EIcircRNAs) and circular intronic RNAs (ciRNAs) (Fig. 1). EcircRNAs only contain exons, and most ecircRNAs are located in the cytoplasm. Two models have been proposed to explain the production of ecircRNAs (Fig. 1A) (Chen, 2016; Wu et al., 2017a; Kristensen et al., 2019; Xiao et al., 2020). The first model suggests that when the pre-mRNA is partially folded during transcription, canonical splicing will cause an “exon jump” and generate a linear RNA containing skipped exons (Starke et al., 2014; Kelly et al., 2015). A subsequent back-splicing event will turn the ‘lariat intermediate’ into a closed circular transcript (Barrett et al., 2015; Li et al., 2018d). The second model proposes that when the 5-prime ss is pulled closer to the 3-prime ss by base pairing of flanking intronic complementary sequences (Ivanov et al., 2014; Vicens and Westhof, 2014; Zhang et al., 2014; Wilusz, 2015) or specific bindings of intronic sequences to RBPs (Conn et al., 2015), a back-splicing event may occur, and a circRNA may be generated (Jeck et al., 2013). These models are supported by accumulating evidence showing that cis-acting elements (Jeck et al., 2013; Zhang et al., 2014; Yoshimoto et al., 2019) and trans-acting factors (Ashwal-Fluss et al., 2014; Conn et al., 2015; Agirre et al., 2019) play crucial regulatory roles in circRNA formation. EIcircRNAs are generated when an intron or several introns are alternatively retained in splicing (Li et al., 2015c). Therefore, EIcircRNAs are mainly considered intermediates of ecircRNAs (Li et al., 2015c). CiRNAs, which only contain introns, are produced through escape from debranching of intron lariats (Zhang et al., 2013). This process depends on a GU-rich motif near the 5-prime ss and a C-rich motif close to the branch-point site (Zhang et al., 2013). Unlike ecircRNAs, EIcircRNAs and ciRNAs are mainly located in the nucleus.

## THE FUNCTION OF CIRCRNAS

With the rapid application of high-throughput RNA-sequencing (RNA-seq) technology (Stark et al., 2019) and related bioinformatics tools that identify circRNAs from RNA-seq datasets (Jeck and Sharpless, 2014; Szabo and Salzman, 2016; Li et al., 2017a; Gao and Zhao, 2018), more than one million circRNAs have been annotated in model organisms (Glažar et al., 2014; Vo et al., 2019; Cai et al., 2020; Wu et al., 2020a). However, the functional characterization of circRNAs is still at its early stage (Chen, 2016, 2020; Li et al., 2018d). Their circular conformation and the large sequence overlap with their linear mRNA counterparts have posed substantial challenges in investigating the functions of circRNAs (Li et al., 2018d). Based on our current knowledge, circRNAs have diverse functions as miRNA decoys, protein regulators and translation templates (Fig. 1B, see (Chen, 2016, 2020; Li et al., 2018d) for excellent reviews).

### MiRNA decoys

The most well-known function of circRNAs is that they can act as a sponge to inhibit the function of miRNAs and indirectly regulate the expression of miRNA target genes at the posttranscriptional level (Fig. 1B) (Hansen et al., 2013; Memczak et al., 2013; Weng et al., 2017). For example, *Cdr1as* can compete with mRNAs by binding *miR-7* through miRNA response elements (MREs) (Hansen et al., 2013). A follow-up *in vivo* experiment that knocked down the *Cdr1as* locus in the mouse genome confirmed the importance of the *Cdr1as* and *miR-7* interaction in normal brain function (Piwecka et al., 2017). Although it may not be a common phenomenon for most circRNAs (Guo et al., 2014), several abundant circRNAs also function as miRNA sponges, including *circASAP1* (Hu et al., 2019c), *circBIRC6* (Yu et al., 2017), *circHIPK2* (Huang et al., 2017b), *circHIPK3* (Zheng et al., 2016) and *circSry* (Hansen et al., 2013).

### Protein regulators

In addition to miRNAs, circRNAs can interact with proteins or protein complexes to regulate protein expression and function (Fig. 1B) (Huang et al., 2020a). First, circRNAs can bind to some RBPs and act as RBP sponges. *CircMbl*, a circRNA originating from the second exon of the splicing factor muscleblind (*MBL*), is the first circRNA that was discovered to function as an RBP sponge (Ashwal-Fluss et al., 2014). It was observed that both the circular exon and the flanking introns of *circMbl* contain many *MBL* binding sites that can be specifically bound by *MBL* (Ashwal-Fluss et al., 2014). Therefore, increased expression of *MBL* can promote back-splicing of *circMbl* and lead to decreased expression of canonical linear *MBL* transcripts. At the same time, *circMbl* can sequester *MBL* in the cytoplasm and prevent it from performing splicing functions (Ashwal-Fluss et al., 2014). Several other circRNAs, such as *circAmotl1* (Yang et al., 2017b), *circANRIL* (Holdt et al., 2016), *circPABPN1* (Abdelmohsen et al., 2017), *circPOLR2A* (Li et al., 2017e) and *circDHX34* (Li et al., 2017e), can function as protein decoys as well. Second, circRNAs can form a complex with other RNAs and proteins to perform functions. For example, some intron-containing circRNAs, such as *circEIF3J* and *circBPTF*, can interact with U1 snRNP to form an RNA-protein complex, which further interacts with RNA polymerase II (*Pol II*) at the promoter region of their parental genes and enhances their transcription (Zhang et al., 2013; Li et al., 2015c). Similarly, some ciRNAs, such as *ci-ankrd52*, can also associate with *Pol II* as well (Zhang et al., 2013; Li et al., 2015c). They can also regulate the expression of their parental gene by modulating the elongation of *Pol II* (Zhang et al., 2013; Li et al., 2015c). Many circRNAs, rather than a specific circRNA, can interact with *NF90*/*NF110* as a group to form circRNP complexes in the cytoplasm, which may function as a reservoir of *NF90*/*NF110* (Li et al., 2017e). Upon viral invasion, *NF90*/*NF110* could be released from circRNP complexes and bind to viral mRNAs to perform its antiviral functions (Li et al., 2017e). Third, the interaction of circRNAs with proteins can facilitate or block the function of their binding proteins. For instance, increased expression of *circFoxo3* in the cytoplasm may arrest the anti-senescent protein *ID1*, transcription factor *E2F1*, anti-stress proteins *FAK* and *HIF1α* and prevent the nuclear translocation of these transcription factors and their function, thus leading to increased cellular senescence (Du et al., 2017). *CircFoxo3* can also form a tertiary circRNA-protein complex with cyclin-dependent kinase 2 (*CDK2*) and cyclin-dependent kinase inhibitor 1 (*p21*), which facilitates the inhibition of *CDK2* by *p21*, thus blocking cell cycle progression (Du et al., 2016a). Moreover, *circFoxo3* can bind to *Mdm2* and *p53* in a breast cancer cell line, which leads to tumor cell apoptosis (Du et al., 2016b).

### Translation

Some circRNAs with an internal ribosome entry site (IRES), such as *circMbl* (Pamudurti et al., 2017) and *circZNF609* (Legnini et al., 2017), can share the start codon of their host genes and be translated in a cap-independent manner. The translation initiation of circRNAs may be promoted by N6-methyladenosine (m^6^A) modification of circRNAs (Yang et al., 2017c). In the human heart, 40 circRNAs were observed to be associated with ribosomes and thus may be translated (Heesch et al., 2019). Some of them, including *circSLC8A1*, *circMYBPC3* and *circRYR2*, are heart-specific circRNAs (Heesch et al., 2019). The translation of these circRNAs may be related to the physiological roles of the human heart. Further functional analysis observed that some circRNA-encoded proteins can exert agonist or antagonist effects on cancer progression. For example, *PINT87aa* (Zhang et al., 2018d) and *AKT3-A74aa* (Xia et al., 2019), two peptides encoded by circRNAs, can interact with signal factors and inhibit glioblastoma tumorigenicity in glioblastoma. However, *circGprc5a*-secreted peptides can promote bladder oncogenesis and metastasis through its binding to *Gprc5a* (Gu et al., 2018).

## CIRCRNA EXPRESSION AND ITS IMPLICATION IN DISEASES

Given the important functions that circRNAs can perform, their expression may provide significant clues in understanding the underlying mechanisms of biological processes and disease states. Previous studies have observed that circRNAs are widely expressed in all tissues (Zhou et al., 2018a; Ji et al., 2019; Wu et al., 2020a) and cell types (Salzman et al., 2013) of nearly all species across the eukaryotic tree of life (Wang et al., 2014). Specifically, circRNAs are enriched in brain samples (Westholm et al., 2014; Rybak-Wolf et al., 2015; Szabo et al., 2015; Venø et al., 2015) and human blood samples (Memczak et al., 2015), including peripheral blood mononuclear cells (PBMCs) (Qian et al., 2018), erythrocytes (Alhasan et al., 2016), platelets (Alhasan et al., 2016) and exosomes (Li et al., 2015b). Furthermore, circRNAs are expressed in tissue- (Guo et al., 2014; Szabo et al., 2015; Zhou et al., 2018a) or cell-type- (Salzman et al., 2013) specific and age-dependent manner (Rybak-Wolf et al., 2015; Szabo et al., 2015; You et al., 2015; Zhou et al., 2018a). Using the rat *BodyMap* dataset, we observed tissue-specific circRNA expression in all 11 rat tissues, which may be closely related to the physiological functions of those tissues (Zhou et al., 2018a). The dynamic expression of circRNAs across time has also been correlated with neuronal differentiation (Rybak-Wolf et al., 2015), neural development (Szabo et al., 2015; You et al., 2015), human terminal B cell differentiation (Agirre et al., 2019) and spermatogenesis (Zhou et al., 2018a).

The spatiotemporal expression of circRNAs is mediated by the balance between circRNA generation and circRNA turnover, which can be regulated at both the transcriptional and posttranscriptional levels (Li et al., 2018d; Chen, 2020). On the one hand, the production of circRNAs can be regulated by intronic complement sequences (ICSs) (Jeck et al., 2013; Liang and Wilusz, 2014; Zhang et al., 2014), cis-splicing elements (Ashwal-Fluss et al., 2014; Starke et al., 2014; Wang and Wang, 2015) and trans-acting splicing factors (Ashwal-Fluss et al., 2014; Conn et al., 2015; Kramer et al., 2015; Khan et al., 2016; Aktaş et al., 2017; Errichelli et al., 2017; Fei et al., 2017; Li et al., 2017e). On the other hand, the degradation of circRNAs can be affiliated by miRNA-initiated *AGO2* cleavage (Kleaveland et al., 2018) and nuclease-mediated cleavage, including RNase L endonuclease (Liu et al., 2019a), RNase P/MRP endonuclease complex (Park et al., 2019) and *G3BP1* endonuclease (Fischer et al., 2020). Dysregulation of circRNA generation or turnover may lead to aberrant circRNA expression in cells or tissues, which may be related to many human diseases (Chen et al., 2016). *Cdr1as*, one of the most studied circRNAs, harbors more than 70 binding sites for *miR-7*, and its overexpression strongly inhibits the activity of the tumor suppressor *miR-7* (Hansen et al., 2013). Accumulating evidence has demonstrated its significant increase in colorectal cancer samples, which can inhibit *miR-7* function and activate *EGFR* and *RAF1* oncogenes (Weng et al., 2017). Moreover, the dysregulation of the *miR-7*/*Cdr1as* axis is involved in some other diseases, such as Alzheimer’s diseases (Lukiw, 2013), diabetes (Xu et al., 2015) and cardiometabolic diseases (Geng et al., 2016)*.* Furthermore, the loss of the *Cdr1as* locus causes miRNA deregulation and affects synaptic transmission in knockout mice (Piwecka et al., 2017). In addition to *Cdr1as*, other circRNAs are also implicated in many different human diseases, including cancers (Zhang et al., 2018j; Chen et al., 2019b; Smid et al., 2019; Su et al., 2019; Vo et al., 2019), cardiovascular diseases (Zhang et al., 2018j; Aufiero et al., 2019), neurodevelopment and neurodegenerative diseases (Kumar et al., 2017; Zhang et al., 2018j; Dube et al., 2019; Chen et al., 2020d; Hanan et al., 2020) and immune diseases (Chen et al., 2019c; Zhou et al., 2019b).

## CIRCRNAS ARE PROMISING BIOMARKERS FOR HUMAN DISEASES

In general, biomarkers are physiological indexes or biological molecules that can be objectively measured and can indicate either a normal or a pathogenic state (Lesko and Atkinson, 2001). Technological advances in genomics, transcriptomics, proteomics and metabolomics have led to the discovery and validation of many biomarkers that are pushing personalized medicine forward (Chen et al., 2012; Karczewski and Snyder, 2018; Ahadi et al., 2020). A good biomarker with clinical significance must meet the criteria of analytical validity, clinical validity and clinical utility (Byron et al., 2016). As a novel type of noncoding RNA, circRNA has several distinct advantages over canonical linear RNAs as a disease biomarker (Zhang et al., 2018j). First, circRNA is more stable than linear RNAs because it has a closed loop structure without 5-prime and 3-prime ends (Enuka et al., 2015; Li et al., 2015b). In the *MCF10A* human mammary epithelial cell line, Enuka et al. observed at least 2.5 times longer half-lives of circRNAs compared to linear RNAs, including mRNAs and miRNAs (Enuka et al., 2015). Second, circRNAs are abundantly expressed in many tissue samples. For example, brain samples express more than 10,000 circRNAs in the rat *BodyMap* dataset (Zhou et al., 2018a). In some cases, the expression values of circRNAs are much higher than their linear counterparts (Westholm et al., 2014; Rybak-Wolf et al., 2015; You et al., 2015; Liang et al., 2017). Third, circRNAs are expressed in a tissue-specific (Salzman et al., 2013; Guo et al., 2014; Szabo et al., 2015; Zhou et al., 2018a) and developmental stage-specific manner (Rybak-Wolf et al., 2015; Szabo et al., 2015; You et al., 2015; Zhou et al., 2018a). Importantly, the tissue specificity of circRNA is higher than that of mRNA of its host gene (Guo et al., 2014; Zhou et al., 2018a). These features suggest that circRNAs may have better analytical validity (Zhang et al., 2018j), including analytical specificity, robustness, reproducibility and repeatability (Byron et al., 2016), when used as biomarker molecules. In a recent study, Maass et al. investigated circRNA expression in 20 clinically relevant tissue samples, which underscored the feasibility of using circRNAs as potential disease biomarkers (Maass et al., 2017).

To date, many circRNAs have been identified as biomarkers for human diseases (Zhang et al., 2018j), especially human cancers (Li et al., 2015a; Meng et al., 2017; Su et al., 2019; Sheng et al., 2020). For example, *Cdr1as* has been revealed as a prognostic biomarker in colorectal cancer patients (Weng et al., 2017). In a training cohort comprising 153 primary colorectal cancer tissues and 44 matched normal mucosae, significantly increased *Cdr1as* expression was observed in colorectal cancer tissues, and its overexpression was associated with poor patient survival. The prognostic power of *Cdr1as* was further validated in an independent validation cohort comprising 165 colorectal cancer patients (Weng et al., 2017). A four-circRNA signature, consisting of *hsa_circ_101308*, *hsa_circ_104423*, *hsa_circ_104916* and *hsa_circ_100269*, has been constructed that can predict the early recurrence of stage III gastric cancer (GC) patients after surgery (Zhang et al., 2017). Moreover, Vo et al. constructed a comprehensive catalog of circRNAs in human cancer tissues in a systematic analysis of more than 2,000 cancer samples (Vo et al., 2019). They identified two circRNAs, *circ-AURKA* and *circ-AMACR*, as potential diagnostic biomarkers for neuroendocrine prostate cancer (Vo et al., 2019). Although these circRNA biomarkers are potentially useful in cancer management, most of them are derived from tissue samples (Li et al., 2015a; Meng et al., 2017; Su et al., 2019; Sheng et al., 2020). To improve biomarker accessibility, especially for biomarkers of cancer screening and diagnosis, circRNA biomarkers in body fluids are ideal for clinical application (Anfossi et al., 2018). Hence, the authors further investigated and validated the reliability of detecting circRNA biomarkers in urine samples of prostate cancer patients (Vo et al., 2019), which suggests that circRNA in urine samples is a promising strategy for prostate cancer screening.

## BLOOD CIRCRNAS AND LIQUID BIPOSY BIOMARKERS

Liquid biopsy has been a revolutionary tool in disease management, supporting the diagnosis, prognosis and treatment guidance of human diseases (Heitzer et al., 2019; Mattox et al., 2019; Rubis et al., 2019; Luo et al., 2020a). In comparison to urine, saliva or cerebrospinal fluid, peripheral blood has been used as the major body fluid in liquid biopsy (Rubis et al., 2019; Luo et al., 2020a). In the circulating blood, aberrantly expressed RNAs or fusion transcripts in different blood components have been associated with human cancers (Byron et al., 2016; Zaporozhchenko et al., 2018; Sole et al., 2019) and infectious diseases (Byron et al., 2016; Correia et al., 2017). These RNA biomarkers include cfRNAs in plasma or serum (Mitchell et al., 2008; Fehlmann et al., 2020), exosome-derived RNAs (Maas et al., 2017), EV-incorporated cfRNAs (Maas et al., 2017) and RNA transcripts in tumor-educated platelets (Best et al., 2017, 2018). These cell-free or EV-incorporated RNA biomarkers represent the changes in expression that occur in abnormal cells, such as dysregulated genes in cancer cells (Byron et al., 2016; Rubis et al., 2019; Luo et al., 2020a). Other than cfRNAs, gene expression profiles of PBMCs or whole blood have been proven to be good indicators of many human diseases (Chaussabel and Baldwin, 2014; Chaussabel, 2015), since they can assess the immune status (Chaussabel, 2015). Unlike the expression of cfRNAs in serum, plasma or EVs, RNA expression levels in PBMCs or whole blood are measures of the host response to exogenous pathogens or autoantigens (Schnell et al., 2018; Shaked, 2019). Several whole blood or PBMC gene expression signatures have been developed for cancer management, including the early diagnosis of colorectal cancer (Marshall et al., 2010; Ciarloni et al., 2015, 2016) and lung cancer (Showe et al., 2009; Kossenkov et al., 2018), the prognosis of adult acute myeloid leukemia (Bullinger et al., 2004; Valk et al., 2004) and prostate cancer patients (Ross et al., 2012), and the monitoring of renal cell carcinoma relapse (Giraldo et al., 2017). Moreover, whole blood or PBMC RNA signatures have been widely investigated as liquid biopsy diagnostic tools for infectious diseases (Ramilo and Mejias, 2017), such as discriminating influenza from other respiratory viral infections (Zaas et al., 2009), differentiating viral and bacterial infections (Tsalik et al., 2016), diagnosing septic patients (Sweeney et al., 2018; Gunsolus et al., 2019; Mayhew et al., 2020), and discriminating active tuberculosis (TB) patients from patients with latent TB or other lung diseases (Bloom et al., 2013; Anderson et al., 2014; Qian et al., 2016; Zak et al., 2016; Sambarey et al., 2017; MacLean et al., 2019; Warsinske et al., 2019; Esmail et al., 2020; Gupta et al., 2020). In addition, the transcriptome of blood cells has been implicated in assessing the likelihood of developing obstructive coronary artery disease in symptomatic nondiabetic patients (Vargas et al., 2013) and predicting antibody-mediated kidney allograft rejection in kidney transplant patients (Loon et al., 2019).

With regard to RNA molecules, both protein-coding mRNAs (Byron et al., 2016; Sole et al., 2019) and several classes of noncoding RNAs (Byron et al., 2016; Anfossi et al., 2018; Pardini et al., 2019; Sole et al., 2019) have been used as blood disease biomarkers. In comparison to mRNAs or long noncoding RNAs, small noncoding RNAs, such as miRNAs (Mitchell et al., 2008; Max et al., 2018; Fehlmann et al., 2020) and noncanonical small RNAs (Fritz et al., 2016; Pardini et al., 2019), have the advantage of high stability in the circulating blood (Mitchell et al., 2008; Anfossi et al., 2018). This superiority is especially important when translating RNA biomarkers into clinical practice (Byron et al., 2016; Anfossi et al., 2018), since fast mRNA degradation in blood sample processing may affect the performance of mRNA biomarkers (Dvinge et al., 2014). Given the high stability of circRNAs, great efforts and substantial progress have been made to investigate the possibility of using circRNA biomarkers in liquid biopsy in recent years.

**Figure 2:**
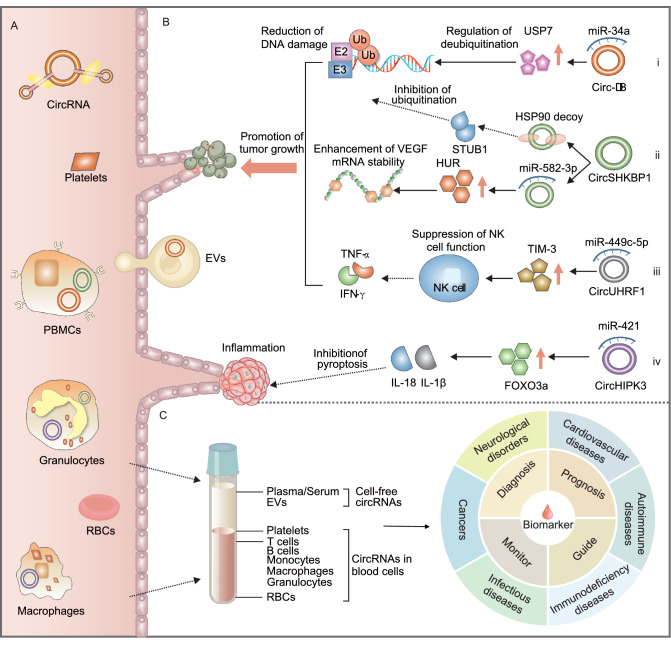
**Peripheral blood circRNAs are implicated in human diseases and can be used as potential disease biomarkers in liquid biopsy.** (A) Peripheral blood circRNAs are abundantly expressed and can be reliably detected in cell-free circulating blood components (such as exosomes, EVs, plasma, and serum) and blood cells (including PBMCs, macrophages, RBCs, and platelets). (B) Some exosomal circRNAs, such as *circ-DB* (i), *circSHKBP1* (ii), and *circUHRF1* (iii), are important regulators in oncogenic pathways, while some circRNAs in exosomes, such as *circHIPK3* (iv), play key roles in the release of inflammatory cytokines. (C) Peripheral blood circRNAs, both cell-free circRNAs and intracellular circRNAs in blood cells, have potential clinical applications as liquid biopsy biomarkers in many human diseases, such as the diagnosis, prognosis and treatment guidance of many human diseases, including autoimmune diseases, cancers, cardiovascular diseases, immuno-deficiency diseases, infectious diseases, and neurodegenerative diseases

Next, we summarize circRNAs in the peripheral blood, their correlations with human diseases and their potential application in liquid biopsy as disease biomarkers (Fig. 2). We classified blood circRNAs into blood cell-free circRNAs and circRNAs in blood cells since they have distinct biological meanings in the context of human diseases. Blood cell-free circRNAs, including circulating cell-free circRNAs in plasma, serum and blood EVs, are secreted from different tissue cells into the blood. Therefore, cell-free circRNAs have corresponding tissue origins, and cell-free circRNA biomarkers represent their clinical significance in the original tissue (Li et al., 2019d, 2020). On the other hand, blood cell circRNAs consist of circRNAs in various blood cells, such as monocytes, erythrocytes, neutrophils and platelets. CircRNAs in mixtures of different blood cells, such as circRNAs in lymphocytes, PBMCs and whole blood, are classified in this group as well. The circRNA expression profiles of peripheral blood cells or a mixture of blood cells are important indicators of the host’s immune status (Chaussabel, 2015), which may undergo dynamic changes during acute events such as viral infections (Chen et al., 2018a; Rose et al., 2019; Zhou et al., 2019a). Hence, circRNA biomarkers in blood cells, PBMCs or whole blood represent the specific immune response of an individual to different physiological aspects.

### Cell-free circRNAs in peripheral blood

Mounting evidence has demonstrated abundant circRNA expression in blood plasma (Maass et al., 2017; Yi et al., 2018; Smid et al., 2019) and serum (Gu et al., 2017; Maass et al., 2017; Sonnenschein et al., 2019; Sun et al., 2020b) (Fig. 2A). In the circulating blood, EVs, including exosomes and microvesicles, are important blood components that have great diagnostic and prognostic value (Revenfeld et al., 2014). Li et al. investigated circRNA expression in *MHCC-LM3* liver cancer cells and cell-derived exosomes and found at least 2-fold circRNA enrichment in exosomes compared to producer cells (Li et al., 2015b). They further found that the expression level of exosomal circRNAs is largely the same after serum incubation at room temperature for up to 24 h (Li et al., 2015b). Using the extracellular vesicle long RNA (exLR) sequencing method, the same group explored the expression profiles of circRNAs in 352 plasma EV samples (Li et al., 2019d). They found 137,196 circRNA candidates that were expressed in normal plasma EV samples, and the circular to linear ratio was significantly higher in EVs than in PBMCs (Li et al., 2019d). A recent study also isolated platelet-derived EVs and demonstrated the selective release of platelet-specific circRNAs in exosomes and microvesicles (Preußer et al., 2018). Based on these data, a database of blood exosomal circRNAs, exoRBase, has been developed to facilitate the development of circRNA signatures in blood exosomes (Li et al., 2017b). In addition, two previous studies have observed that the majority of circulating cell-free miRNAs were associated with *AGO2* protein complexes rather than with vesicles in blood plasma (Arroyo et al., 2011; Turchinovich et al., 2011). Like cell-free miRNAs, blood cell-free circRNAs may bind to several RNA-binding proteins or protein complexes as well (Huang et al., 2020a). Therefore, some cell-free circRNAs may not be associated with blood EVs. In a pilot study of circRNA expression profiles in 348 primary breast cancer tissue samples, Smid et al. found that *circCNOT2* is a prognostic biomarker of aromatase inhibitor therapy in advanced breast cancer patients (Smid et al., 2019). To investigate the possibility of using *circCNOT2* as a noninvasive biomarker, they further amplified *circCNOT2* in plasma samples of four breast cancer patients and found variable *circCNOT2* expression in all plasma samples (Smid et al., 2019). All these results suggest that cell-free circRNAs in peripheral blood are stable, enriched and detectable.

Blood EVs can be secreted from cells of many different biological systems and can be circulated to recipient cells through the bloodstream (Revenfeld et al., 2014; Lasda and Parker, 2016). Therefore, blood cell-free circRNAs can perform vital roles in many biological processes, such as cancer cell proliferation, cancer metastasis, drug resistance, hemostasis and inflammation (Fig. 2B) (Wang et al., 2019e; Cui et al., 2020; Li et al., 2020; Shang et al., 2020; Xu et al., 2020b). For example, *circ-deubiquitination* (*circ-DB*) in adipose-secreted exosomes was found to regulate deubiquitination via the suppression of *miR-34a* and the activation of deubiquitination-related *USP7* in plasma samples of hepatocellular carcinoma (HCC) patients, which could reduce DNA damage and promote HCC cell growth (Zhang et al., 2018b). Exosomal *circSHKBP1* was found to promote the progression of GC by regulating the *miR-582-3p*/*HUR*/*VEGF* pathway and suppressing *HSP90* degradation (Xie et al., 2020a). *CircUHRF1* in plasma exosomes can inhibit the functions of natural killer cells by upregulating the expression of *TIM-3* via *miR-449c-5p* degradation and drive resistance to anti-*PD1* immunotherapy in HCC patients (Zhang et al., 2020a). Moreover, exosomal *circHIPK3* can prevent ischemic muscle injury by downregulating *miR-421* expression, increasing *FOXO3a* expression, inhibiting pyroptosis and releasing *IL-1β* and *IL-18* (Yan et al., 2020).

Given the biological functions that blood cell-free circRNAs can perform (Wang et al., 2019e; Cui et al., 2020; Li et al., 2020) and their implications in many human diseases, cell-free circRNAs in the peripheral blood have great potential as liquid biopsy biomarkers of human diseases (Fig. 2C) (Anfossi et al., 2018; Zaporozhchenko et al., 2018; Zhang et al., 2018j). To date, many blood cell-free circRNAs have been introduced for cancer management, including early cancer diagnosis, cancer prognosis and prediction of cancer treatment (Preußer et al., 2018; Aufiero et al., 2019; Fraipont et al., 2019; Lu et al., 2019c; Pardini et al., 2019; Sole et al., 2019; Su et al., 2019; Wang et al., 2019e; Beltrán-García et al., 2020; Cui et al., 2020; Li et al., 2020). In a recent study, Luo et al. measured the expression levels of two circRNAs in plasma samples of 231 lung cancer patients and 41 healthy controls using reverse transcription droplet digital PCR (RT-ddPCR) (Luo et al., 2020c). They identified *hsa_circ_0000190* as a circRNA biomarker in human blood plasma that can predict the survival outcomes of lung cancer patients (Luo et al., 2020c). Furthermore, the increased expression of plasma *hsa_circ_0000190* was also correlated with poor response to systemic therapy and immunotherapy in lung cancer patients (Luo et al., 2020c). Similarly, Li et al. investigated the clinical relevance of serum exosomal *circFLI1* in lung cancer patients in a cohort of 61 small cell lung cancer (SCLC) patients and 55 normal subjects. They found that serum exosomal *circFLI1* levels were significantly higher in SCLC patients, especially in SCLC patients with distant metastasis (Li et al., 2018b). Notably, they observed that SCLC patients with lower exosomal *circFLI1* expression levels experienced longer disease remissions, indicating its prognostic power in SCLC. The authors also suggested that serum exosomal *circFLI1* may be used as a biomarker that can monitor the clinical response to chemotherapy in SCLC patients (Li et al., 2018b). By analyzing blood plasma samples of 62 GC patients and 25 healthy controls, Tang et al. proposed a novel circulating diagnostic biomarker of GC, plasma exosomal *circKIAA124*, that was correlated with clinical TNM stage, lymphatic metastasis and overall survival time of GC patients (Tang et al., 2018). In addition to the aberrant expression of blood cell-free circRNAs, the presence of some fusion circRNAs (f-circRNAs) in the peripheral blood has also been used as a liquid biopsy biomarker. Guarnerio et al. found that f-circRNAs could be produced from cancer-associated chromosomal translocations in cancer cells (Fig. 1), and f-circRNAs could promote cellular transformation, cell viability and resistance upon therapy (Guarnerio et al., 2016). In a systematic analysis of f-circRNAs in localized prostate cancer tissues, Chen et al. observed more f-circRNAs in tumors with worse prognosis (Chen et al., 2019b). Due to the lack of recurrence of these f-circRNAs, the authors suggested that f-circRNAs are good biomarker candidates (Chen et al., 2019b). In NSCLC patients, Tan et al. found that *F-circEA*, an f-circRNA originating from the *EML4-ALK* fusion gene, was exclusively expressed in the plasma of patients with the *EML4-ALK* fusion (Tan et al., 2018). Therefore, plasma *F-circEA* may serve as a liquid biopsy biomarker to diagnose NSCLC patients with *EML4-ALK* translocation and guide targeted therapy for NSCLC patients in this subgroup (Tan et al., 2018). More cell-free circRNA biomarkers in the blood are summarized in Table 1.

**Table 1:** Cell-free circRNA biomarkers in circulating peripheral blood.

**Disease**	**CircRNA biomarker**	**Source**	**Expression change**	**Cohort size**	**Clinical significance**	**AUC**	**Method**	**Reference**
Breast cancer	Hsa_circ_0001785	Plasma	Up	57 breast cancer/17 HC	Associated with histological grade, TNM stage and distant metastasis; significant expression difference between pre-treatment and post-treatment.	0.784	Microarray RT-qPCR	Yin et al. (2018)
Bladder cancer	Hsa_circ_0000285	Serum	Down	97 Bladder cancer/97 HC	Associated with tumor size, differentiation, LNM, distant metastasis, TNM stage and cisplatin response.	NA	RT-qPCR	Chi et al. (2019)
CA	Hsa_circ_0003204	Plasma derived EV	Up	35 CA/32 HC	Associated with proliferation, migration and tube formation of endothelial cells.	0.770	RT-qPCR	Zhang et al. (2020b)
CAD	Hsa_circ_0005540	Plasma derived exosome	Up	108 CAD/89 HC	Associated with the Framingham Heart Study risk factors.	0.853	RNA-seq RT-qPCR	Wu et al. (2020b)
CHB	CircMTO1	Serum	Down	360 CHB/360 HC	Associated with liver fibrosis progression and prognosis.	0.914	RT-qPCR	Wang et al. (2019c)
CHD	Hsa_circ_004183 Hsa_circ_079265 Hsa_circ_105039	Plasma	Down	40 CHD/40 HC	NA	0.965	Microarray RT-qPCR	Wu et al. (2019a)
CLL	Circ-RPL15	Plasma	Up	150 CLL/65 HC	Associated with progression and outcome.	0.840	Microarray RT-qPCR	Wu et al. (2020c)
CRC	Hsa_circ_0006990	Plasma	Up	60 CRC/43 HC	Associated with TNM stage.	0.724	RT-qPCR	Li et al. (2019c)
CRC	Circ-CCDC66 Circ-ABCC1 Circ-STIL	Plasma	Down	45 CRC/61 HC	Circ-ABCC1 was associated with tumor growth and progression; significant expression difference of circ-CCDC66 between pre-treatment and post-treatment.	0.780	RT-qPCR	Lin et al. (2019)
CRC	Hsa_circ_0004771	Serum derived exosome	Up	135 CRC/45 HC	Associated with TNM stage and distant metastasis; significant expression difference between pre-treatment and post-treatment.	0.880	RT-qPCR	Pan et al. (2019)
CRC	Hsa_circ_0000826	Serum	Up	100 CRC/100 HC	Associated with liver metastasis.	0.778	RT-qPCR	Shi et al. (2020)
CRC	Hsa_circ_0101802	Serum derived exosome	Up	221 CRC/221 HC	NA	0.854	RNA-seq RT-qPCR	Xie et al. (2020c)
CRC	Hsa_circ_0082182 Hsa_circ_0000370 Hsa_circ_0035445	Plasma	Up Up Down	156 CRC/66 HC	The first two circRNAs were associated with LNM and had significant expression difference between pre-treatment and post-treatment; the third was associated with TNM stage.	0.835	Microarray RT-qPCR	Ye et al. (2019)
CRC	Hsa_circ_0007534	Plasma	Up	112 CRC/46 HC	Associated with progression of clinical classification, metastatic phenotype, and differentiation.	0.780	RT-qPCR	Zhang et al. (2018f)
EC	Hsa_circ_0109046 Hsa_circ_0002577	Serum derived EV	Up	10 EC/10 HC	NA	NA	RNA-seq RT-qPCR	Xu et al. (2018a)
EOC	CircBNC2	Plasma	Down	83 EOC/83 benign ovarian cyst/83 HC	Associated with histological grade, serious subtype, LNM and distant metastasis.	0.923	RT-qPCR	Hu et al. (2019b)
ESCC	Hsa_circ_0001946 Hsa_circ_0043603	Plasma	Down	50 ESCC/50 HC	Associated with recurrence, overall survival and disease-free survival.	0.894 0.836	Microarray RT-qPCR	Fan et al. (2019)
ESCC	CircGSK3ß	Plasma	Up	86 ESCC/11 benign lesion/43 HC	Associated with recurrence, metastasis, clinical stage and outcome; significant expression difference between pre-treatment and post-treatment.	0.793	Microarray ddPCR RT-qPCR	Hu et al. (2019a)
ESCC	Circ-TTC17	Plasma	Up	30 ESCC/25 HC	Associated with TNM stage, LNM and survival time.	0.820	RT-qPCR	Wang et al. (2019b)
ESCC	Circ-SLC7A5	Plasma	Up	87 ESCC/53 HC	Associated with TNM stage and survival time.	0.772	Microarray RT-qPCR	Wang et al. (2020c)
GBM	CircFOXO3 Hsa_circ_0029426 Circ-SHPRH	Plasma	Down	100 GBM/100 HC	NA	0.906-0.980	RT-qPCR	Chen et al. (2020a)
GC	Hsa_circ_0000190	Plasma	Down	104 GC/104 HC	Associated with blood carcinoembryonic antigen level.	0.600	RT-qPCR	Chen et al. (2017c)
GC	Hsa_circ_0021087 Hsa_circ_0005051	Plasma	Down	70 GC/70 HC	Associated with tumor size and TNM stage; significant expression difference of hsa_circ_0021087 between pre-treatment and post-treatment.	0.773	RT-qPCR	Han et al. (2020c)
GC	Hsa_circ_0000745	Plasma	Down	60 GC/60 HC	Associated with TNM stage.	0.683	RNA-seq RT-qPCR	Huang et al. (2017a)
GC	Hsa_circ_00001649	Serum	Down	20 GC (pre-operation vs. post-operation)	Associated with pathological differentiation; significant expression difference between pre-treatment and post-treatment.	0.834	RT-qPCR	Li et al. (2017d)
GC	Hsa_circ_0001017 Hsa_circ_0061276	Plasma	Down	121 GC/121 HC	Associated with distal metastasis, overall survival, prognosis and outcome.	0.912	Microarray RT-qPCR RT-ddPCR	Li et al. (2018c)
GC	Hsa_circ_0000467	Plasma	Up	40 GC/20 HC	Associated with TNM stage; significant expression difference between pre-treatment and post-treatment.	0.790	RT-qPCR	Lu et al. (2019a)
GC	Hsa_circ_0006848	Plasma	Down	30 GC/30 HC	Associated with differentiation and tumor size; significant expression difference between pre-treatment and post-treatment.	0.733	RT-qPCR	Lu et al. (2019b)
GC	Hsa_circ_0010882	Plasma	Up	66 GC/66 HC	Associated with tumor size and histological grade, overall survive and prognosis.	NA	RT-qPCR	Peng et al. (2020)
GC	CircPSMC3	Plasma	Down	106 GC/21 HC	Associated with TNM stage, LNM and overall survival.	0.933	Microarray RT-qPCR	Rong et al. (2019)
GC	Hsa_circ_0065149	Plasma derived exosome	Down	39 GC/41 HC	NA	0.640	RT-qPCR	Shao et al. (2020)
GC	CircKIAA1244	Plasma	Down	62 GC/25 HC	Associated with TNM stage, LNM and overall survival.	0.748	Microarray RT-qPCR	Tang et al. (2018)
GC	Hsa_circ_0000419	Plasma	Down	44 GC/43 HC	Associated with tumor stage, lymphatic and distal metastasis, venous and perineural invasion.	0.840	RT-qPCR	Tao et al. (2020)
GC	Hsa_circ_0000936	Serum derived exosome	Up	32 GC/20 HC	Associated with TNM stage and prognosis; significant expression difference before and after gastrectomy.	NA	RT-qPCR	Xie et al. (2020a)
GC	Hsa_circ_0000181	Plasma	Down	102 GC/105 HC	Associated with differentiation and carcinoembryonic antigen.	0.756	RT-qPCR	Zhao et al. (2018b)
Glioma	CircNF1X	Serum derived exosome	Up	69 glioma/10 HC	Associated with temozolomide response and prognosis.	0.885	RT-qPCR	Ding et al. (2020)
HCC	Hsa_circ_0051443	Plasma derived exosome	Down	60 HCC/60 HC	Associated with progression.	0.809	Microarray RT-qPCR	Chen et al. (2020c)
HCC	Circ-ZEB1.33	Serum	Up	64 HCC/30 HC	Associated with TNM stage and prognosis.	NA	RT-qPCR	Gong et al. (2018)
HCC	Hsa_circ_100338	Serum derived exosome	Up	39 HCC (pre-operation vs. post-operation)	Associated with metastasis, proliferation, angiogenesis and prognosis.	NA	RT-qPCR	Huang et al. (2020b)
HCC	CircSMARCA5	Plasma	Down	135 HCC/143 cirrhosis /117 hepatitis/103 HC	NA	0.938	RT-qPCR	Li et al. (2019e)
HCC	Hsa_circ_0003998	Plasma	Up	100 HCC/50 hepatitis/50 HC	Significant expression difference between pre-treatment and post-treatment.	0.892	RT-qPCR	Qiao et al. (2019)
HCC	Hsa_circ_0004001 Hsa_circ_0004123 Hsa_circ_0075792	Serum	Up	21 HCC/32 HC	Associated with TNM stage and tumor size.	0.890	RT-qPCR	Sun et al. (2020b)
HCC	Circ_FOXP1	Serum	Up	30 HCC/16 HC	Associated with tumor size, microvascular invasion and advanced TNM stage and survival time.	0.932	RT-qPCR	Wang et al. (2020d)
HCC	Hsa_circ_104075	Serum	Up	101 HCC/60 HC/23 hepatitis B/26 hepatitis C/23 cirrhosis/20 LC/19 GC/30 colon cancer/21 breast cancer	Associated with TNM stage; significant expression difference between pre-treatment and post-treatment.	0.973	RT-qPCR	Zhang et al. (2018g)
HCC	Hsa_circ_0001445	Plasma	Down	104 HCC/57 cirrhosis/44 hepatitis B/52 HC	Associated with serum alpha-fetoprotein level.	0.862	RT-qPCR	Zhang et al. (2018h)
HCM	CircDNAJC6 CircTMEM56 CircMBOAT2	Serum	Down	64 HCM/53 HC	Associated with left ventricular outflow tract gradient and thickness of interventricular septum in patients with obstructive HCM.	0.819 0.756 0.738	RT-qPCR	Sonnenschein et al. (2019)
Hypertension	Hsa_circ_0005870	Plasma	Down	54 Hypertension/54 HC	NA	NA	Microarray RT-qPCR	Wu et al. (2017b)
Heart failure	Hsa_circ_0062960	Plasma	Up	30 Heart failure/30 HC	Associated with B-type natriuretic peptide serum levels.	0.838	Microarray RT-qPCR	Sun et al. (2020c)
IPAH	Hsa_circ_0068481	Serum	Up	82 IPAH/82 HC	Associated with heart function, 6-min walk distance, serum N-terminal pro-B-type natriuretic peptide, serum H2S, risk stratification, right heart failure, and death.	0.895	RT-qPCR	Zhang et al. (2019b)
KD	CircANRIL Hsa_circ_0123996	Serum	Down Up	56 KD/56 HC	Associated with multiple clinical laboratory factors; significant expression difference of circANRIL between pre-treatment and post-treatment.	0.624 0.747	RT-qPCR	Wu et al. (2019b)
LAC	Hsa_circ_0056616	Plasma derived exosome	Up	42 LAC with LNM/48 LAC without LNM	Associated with the level of CXCR4 protein, T stage, M stage, and TNM grade.	0.812	RT-qPCR	He et al. (2020)
LAC	Hsa_circ_0013958	Plasma	Up	30 LAC/30 HC	Associated with TNM stage and LNM.	0.794	Microarray RT-qPCR	Zhu et al. (2017)
LC	Hsa_circ_0000190	Plasma	Up	231 LC/41 HC	Associated with tumor size, histological type, stage, distant metastasis, extrathoracic metastasis, survival, prognosis, PD-L1 level and therapy response.	0.950	RNA-seq RT-qPCR RT-ddPCR	Luo et al. (2020c)
LN	Hsa_circ_002453	Plasma	Up	59 SLE (30 with LN and 29 without LN)/26 RA/32 HC	Associated with severity of renal involvement and 24-hour proteinuria.	0.906	Microarray RT-qPCR	Ouyang et al. (2018)
LUAD	Hsa_circ_0005962 Hsa_circ_0086414	Plasma	Up Down	153 LUAD/54 HC	Hsa_circ_0086414 was associated with EGFR mutations; significant expression difference of hsa_circ_0005962 between pre-treatment and post-treatment.	0.810	RT-qPCR	Liu et al. (2019b)
LUAD	Hsa_circ_002178	Serum derived exosome	Up	120 LUAD/30 HC	Associated with programmed cell death protein 1 (PD1) expression.	0.997	RT-qPCR	Wang et al. (2020b)
MCL	CircCDYL	Plasma	Up	18 MCL/17 HC	Associated with cell proliferation.	0.856	RT-qPCR	Mei et al. (2019)
MDD	CircDYM	Plasma	Down	60 MDD/32 HC	Associated with the scores of the 24-item Hamilton Depression Rating Scale, retardation subscale and treatment response.	0.643	RT-qPCR	Song et al. (2020)
NPC	Hsa_circ_0000285	Serum	Up	150 NPC/100 HC	Associated with tumor size, differentiation, LNM, distant metastasis, TNM stage, survival rate and radiotherapy response.	NA	RT-qPCR	Shuai et al. (2018)
NPC	Hsa_circ_0066755	Plasma	Up	86 NPC/86 HC	Associated with clinical stage.	0.904	RT-qPCR	Wang et al. (2020a)
NSCLC	CircFARSA	Plasma	Up	50 NSCLC/50 HC	NA	0.710	RNA-seq RT-qPCR	Hang et al. (2018)
NSCLC	Hsa_circ_0109320	Plasma	Up	90 NSCLC	Associated with progression-free survival and gefitinib response.	0.805	Microarray RT-qPCR	Liu et al. (2019c)
NSCLC	Hsa_circ_0002130	Serum derived exosome	Up	28 drug-resistance NSCLC/32 drug-sensitive NSCLC	Associated with osimertinib response.	0.792	RT-qPCR	Ma et al. (2020)
Osteosarcoma	Hsa_circ_0000885	Serum	Up	55 osteosarcoma/27 benign bone tumor/25 HC	Associated with clinical prognosis; significant expression difference between pre-treatment and post-treatment.	0.783	RNA-seq RT-qPCR	Zhu et al. (2019a)
PBC	Hsa_circ_402458	Plasma	Up	35 PBC/36 HC	NA	0.710	Microarray RT-qPCR	Zheng et al. (2016)
PC	Circ-LDLRAD3	Plasma	Up	31 PC/31 HC	Associated with CA19-9, N classification, venous invasion, lymphatic invasion, stage, metastasis.	0.670	RT-qPCR	Yang et al. (2017a)
PDAC	Hsa_circ_0036627	Plasma derived exosome	Up	93 PDAC/20 HC	Associated with duodenal invasion, vascular invasion, T factor, TNM stage and survival rate.	NA	Microarray RT-qPCR	Li et al. (2018e)
PDAC	Circ-IARS	Plasma derived exosome	Up	20 PDAC with metastasis/20 PDAC without metastasis	Associated with tumor vessel invasion, liver metastasis, TNM stage, and prognosis.	NA	Microarray RT-qPCR	Li et al. (2018a)
PoAF	Hsa_circ_025016	Plasma	Up	769 underwent off-pump coronary artery bypass grafting/15 HC	Associated with fasting blood glucose.	0.802	Microarray RT-qPCR	Zhang et al. (2018c)
SAI	CircFUNDC1	Plasma	Up	26 AIS with SAI/42 AIS without SAI	Associated with neutrophils counts, white blood cell and neutrophil ratios.	0.661	RT-qPCR	Zuo et al. (2020)
SCC	CircFoxO3a	Serum	Down	103 SCC/30 HC	Associated with stromal invasion, LNM and prognosis.	NA	RT-qPCR	Tang et al. (2020)
SCLC	CircFLI1	Serum derived exosome	Up	61 SCLC/55 HC	Associated with tumor survival and chemotherapy response.	NA	RT-qPCR	Li et al. (2018b)
SLE	Hsa_circ_407176 Hsa_circ_001308	Plasma	Down	126 SLE/102 HC	NA	0.599 0.662	Microarray RT-qPCR	Zhang et al. (2018e)
SOC	CircSETDBI	Serum	Up	60 SOC/60 HC	Associated with clinical stage, LNM, chemotherapy response and progression-free survival.	0.830	RT-qPCR	Wang et al. (2019d)
TB	Hsa_circ_0001204 Hsa_circ_0001747	Plasma	Down	195 TB/50 pneumonia/50 LC/50 COPD/170 HC	Associated with the radiological severity scores; significant expression difference between pre-treatment and post-treatment.	0.928	RT-qPCR	Huang et al. (2018b)
TB	Hsa_circ_0001953 Hsa_circ_0009024	Plasma	Up	123 TB/103 HC	Associated with TB severity; significant expression difference between pre-treatment and post-treatment.	0.915	Microarray RT-qPCR	Huang et al. (2018a)
TB	Hsa_circ_051239 Hsa_circ_029965 Hsa_circ_404022	Serum	Up	131 TB/50 pneumonia/53 HC	Hsa_circRNA_051239 was associated with TB drug response.	0.992	Microarray RT-qPCR	Liu et al. (2020)
TB	Hsa_circ_103571	Plasma	Down	35 TB/32 HC	NA	0.838	Microarray RT-qPCR	Yi et al. (2018)
UCB	CircPRMT5	Serum derived exosome	Up	71 UCB/50 HC	Associated with LNM and tumor progression.	NA	RT-qPCR	Chen et al. (2018b)

**Abbreviation:** AIS: acute ischemic stroke; CA: cerebral atherosclerosis; CAD: coronary artery disease; CHB: chronic hepatitis B; CHD: congenital heart diseases; CLL: chronic lymphocytic leukemia; COPD: chronic obstructive pulmonary disease; CRC: colorectal cancer; ddPCR: droplet digital PCR; EC: endometrial cancer; EOC: epithelial ovarian cancer; ESSC: esophageal squamous cell cancer; EV: extracellular vesicle; GBM: glioblastoma; GC: gastric cancer; HC: healthy control; HCC: hepatocellular carcinoma; HCM: hypertrophic cardiomyopathy; IPAH: idiopathic pulmonary arterial hypertension; KD: Kawasaki disease; LAC: lung adenocarcinoma; LC: lung cancer; LN: lupus nephritis; LNM: lymph node metastasis; LUAD: lung adenocarcinoma; MCL: mantle cell lymphoma; MDD: major depressive disorder; NA: not applicable; RT-qPCR: reverse transcription and quantitative PCR; NPC: nasopharyngeal carcinoma; NSCLC: non-small cell lung cancer; PBC: primary biliary cholangitis; PC: pancreatic cancer; PDAC: pancreatic ductal adenocarcinoma; PoAF: postoperative atrial fibrillation; SAI: stroke associated infection; SCC: squamous cervical cancer; SCLC: small cell lung cancer; SLE: systemic lupus erythematosus; SOC: serous ovarian cancer; TB: tuberculosis; TNM: tumor node metastasis; UCB: urothelial carcinoma of the bladder.

### CircRNAs in blood cells and whole blood

CircRNA expression in blood cells and whole blood, a major source of liquid biopsy samples, has been extensively investigated (Fig. 2A). In a pilot study, Memczak et al. detected thousands of circRNAs in peripheral whole blood samples using RNA-seq (Memczak et al., 2015). They found that the expression levels of these blood circRNAs were comparable to those in circRNA-rich cerebellar tissue (Memczak et al., 2015). In separate blood cell populations, circRNAs were observed to be enriched approximately 100-fold in platelets and anucleate erythrocytes relative to nucleated tissues, such as lung, brain and colon (Alhasan et al., 2016). Gaffo et al. investigated circRNA expression in T cells, B cells and monocytes of healthy subjects and found abundant circRNA expression in these mature blood cells (Gaffo et al., 2019). We also explored the expression landscape of circRNAs in PBMCs and found that the expression level of circRNAs in PBMCs, together with that in platelets, red blood cells (RBCs) and whole blood, is high enough to be detected (Qian et al., 2018). All these results suggest that whole blood (Memczak et al., 2015; Qian et al., 2018), PBMCs (Qian et al., 2018), and several blood cells, including neutrophils (Maass et al., 2017), T cells (Gaffo et al., 2019), B cells (Gaffo et al., 2019), monocytes (Gaffo et al., 2019), RBCs (Alhasan et al., 2016; Qian et al., 2018) and platelets (Maass et al., 2017; Qian et al., 2018; Gaffo et al., 2019), are reliable clinical samples for circRNA profiling in liquid biopsy.

**Figure 3:**
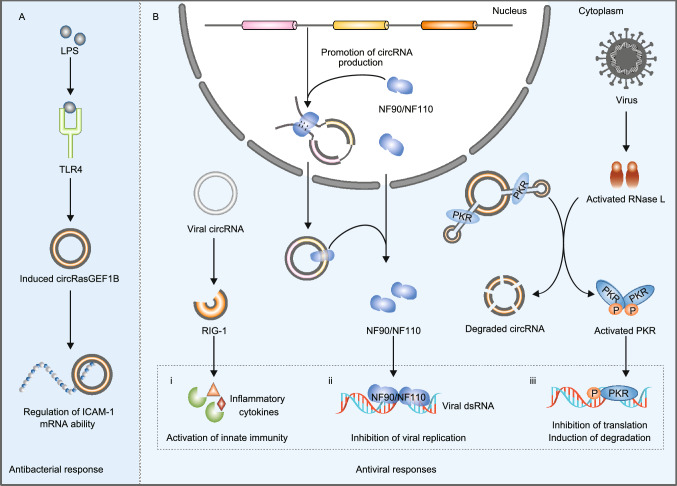
**CircRNAs are actively involved in host immune responses to exogenous pathogens.** (A) *CircRasGEF1B*, a circRNA induced by *LPS*, can protect cells from bacterial infection by regulating the expression of *ICAM-1* mRNA in the *TLR4*/*LPS* pathway. (B) Exogenous circRNAs released by viruses can be recognized by *RIG-I*, thus activating the host innate immunity to viruses (i). Moreover, *NF90*/*NF110* not only promotes circRNA production in the nucleus but also interacts with mature host circRNAs to form circRNP complexes in the cytoplasm. Upon viral infection, *NF90*/*NF110* can be released from circRNP complexes and bind viral mRNAs to inhibit viral replication (ii). In addition, circRNAs can form RNA duplexes and act as inhibitors of *PKR* under normal conditions. When a virus invades the cells of its host, *RNase L* is activated to efficiently degrade circRNAs, and *PKR* is released and activated to initiate the early cellular innate immune response (iii)

Accumulating evidence has suggested that circRNAs play crucial roles in the immune response and its regulation (Fig. 3) (Chen et al., 2017b, 2019c; Xu et al., 2018b, 2020a; Yang et al., 2018; Zhou et al., 2019b; Awan et al., 2020). First, circRNAs are important regulators of blood cell biogenesis, differentiation and activation (Chen et al., 2019c; Zhou et al., 2019b; Xu et al., 2020a). In a comprehensive study of circRNA expression in hematopoietic progenitors, differentiated lymphoid and myeloid cells, Nicolet et al. observed a cell-type specific pattern of circRNA expression profiles in blood cells, and the type and number of circRNAs increased upon hematopoietic maturation (Nicolet et al., 2018). Moreover, Holdt et al. found that *circANRIL* can bind to *PES1* to impede the generation of pre-rRNA and ribosomes, resulting in the biogenesis of macrophages by *p53* activation (Holdt et al., 2016). Second, circRNAs are actively involved in antiviral immune responses (Fig. 3B) (Cadena and Hur, 2017; Awan et al., 2020). For example, Chen et al. showed that exogenous circRNAs released by viruses can be recognized by the pattern recognition receptor *RIG-I* of host cells, which activates host innate immunity (Chen et al., 2017a). In their subsequent work, the authors showed that m^6^A RNA modification of human circRNAs inhibits innate immunity, while unmodified circRNAs and K63-polyubiquitin can activate *RIG-I* and innate immune response (Chen et al., 2019a). Li et al. found that two immune factors, *NF90*/*NF110*, not only promote circRNA production in the nucleus but also bind to mature host circRNAs to form circRNP complexes in the cytoplasm. Upon viral infection, circRNP complexes in the cytoplasm can release *NF90*/*NF110*, which binds viral mRNAs to inhibit viral replication (Li et al., 2017e). Moreover, Liu et al. presented that circRNAs can form RNA duplexes and act as inhibitors of innate immunity-related protein kinase (*PKR*) under normal conditions (Liu et al., 2019a). Upon poly(I:C) treatment or viral infection, RNase L is activated to efficiently degrade circRNAs, and *PKR* is thus released from circRNA inhibition to initiate the early cellular innate immune response (Liu et al., 2019a). Third, circRNAs are closely associated with the antibacterial immune response as well. Ng et al. characterized circRNAs induced by lipopolysaccharide (*LPS*) and identified *circRasGEF1B* as a conserved positive regulator of the *LPS* response (Ng et al., 2016). Their functional analysis revealed that *circRasGEF1B* can induce the expression of *ICAM-1* in the *TLR4*/*LPS* pathway, which activates pathogen recognition and the inflammatory response upon microbial infection (Fig. 3A) (Ng et al., 2016).

The important functions of circRNAs in blood cells suggest that the dysregulation of circRNA expression in blood cells is likely to contribute to the occurrence and progression of immune-related diseases, including autoimmune diseases, infectious diseases and cardiovascular diseases (Chen et al., 2019c; Gaffo et al., 2019; Zhou et al., 2019b; Xie et al., 2020b; Xu et al., 2020a). For instance, we determined that the expression level of PBMC circRNAs is higher in TB patients than healthy controls, and five immune-related pathways were upregulated upon *Mycobacterium tuberculosis* infection, including “cytokine-cytokine receptor interactions”, “chemokine signaling pathways”, and “neurotrophic signaling pathways” (Qian et al., 2018). Similarly, Huang et al. identified 13 upregulated and 24 downregulated circRNAs in PBMCs of TB patients (Huang et al., 2018c). Among them, *hsa_circRNA*_001937 is likely to participate in the inflammatory response by targeting *miR-26b* and modulating the *NF-κB* pathway (Huang et al., 2018c). In addition, Zhang et al. investigated the roles that circRNAs play in early human immunodeficiency virus (HIV) infection (EHI), especially in regulating HIV replication (Zhang et al., 2018i). EHI represents a stage where viral replication increases to a peak level and intense antiviral immune response and immune injury occur (Powers et al., 2011; Richey and Halperin, 2013). The authors characterized the expression profiles of circRNAs, mRNAs and miRNAs in PBMCs of EHI patients and constructed a circRNA-associated competing endogenous network in EHI patients. They revealed 67 differentially expressed circRNAs, such as *CCNK*, *CDKN1A* and *IL-15*, that are potentially involved in HIV replication by regulating the expression of genes in the immune response, inflammatory response and defense response to the virus (Zhang et al., 2018i). Regarding autoimmune diseases, Liu et al. detected a global reduction in circRNAs and activation of RNase L in PBMCs of systemic lupus erythematosus (SLE) patients (Liu et al., 2019a). They further found that *circPOLR2A* overexpression can lead to reduced *PKR* activation, *EIF2α* phosphorylation and type I IFN-induced gene suppression. This highlights the link between circRNAs and innate immunity regulation and provides the potential for circRNA manipulation in SLE treatment (Liu et al., 2019a). In addition to the above examples, the abnormal expression of blood circRNAs is related to several other diseases, such as the immune response to Ebola virus (Wang et al., 2017b) and hepatitis C virus (Jost et al., 2018), rheumatoid arthritis (RA) (Yang et al., 2019), type 2 diabetes mellitus (T2DM) (Fang et al., 2018), heart failure (Han et al., 2020b) and adenosine deaminase deficiency (Maass et al., 2017).

With increasing knowledge of blood cell circRNAs and their function, many circRNAs in blood cells or whole blood have been proposed as liquid biopsy biomarkers for human diseases (Fig. 2C) (Aufiero et al., 2019; Beltrán-García et al., 2020; Kumar et al., 2017; Ravnik-Glavač and Glavač, 2020; Sun et al., 2020a). For instance, we developed a PBMC circRNA-based molecular signature that discriminates active TB patients from healthy controls in our previous study (Qian et al., 2018). The classification power of this PBMC circRNA signature was further validated in an independent cohort with an area under the receiver operating characteristic curve (AUC) of 0.946 (Qian et al., 2018). In another study, Huang et al. found that the expression of *hsa_circ_001937* in PBMCs was significantly higher in TB patients than in pneumonia, chronic obstructive pulmonary disease (COPD) and lung cancer patients (Huang et al., 2018c). In a cohort consisting of 115 TB, 40 pneumonia, 40 COPD, and 40 lung cancer patients and 90 healthy control subjects, *hsa_circ_001937* had good discriminative power with an AUC of 0.873. After anti-TB treatment, the expression level of *hsa_circ_001937* was significantly decreased compared to that of healthy controls. These results suggest that PBMC *hsa_circ_001937* may be a TB diagnostic biomarker (Huang et al., 2018c). Furthermore, Lei et al. found an upregulation of *circ_0000798* expression in PBMCs of HCC patients, which was associated with poor overall survival (Lei et al., 2019). In a cohort of 72 HCC patients and 30 healthy control subjects, *circ_0000798* expression could distinguish HCC patients from healthy controls with an AUC of up to 0.703. The authors suggested that PBMC *circ_0000798* has potential as a blood biomarker for HCC diagnosis and prognosis (Lei et al., 2019). In addition, Li et al. measured circRNA expression changes between children with SLE and healthy children and investigated the significance of blood circRNAs in SLE diagnosis (Li et al., 2019b). They identified and validated the diagnostic power of two circRNAs in whole blood*, hsa_circ_0057762* and *hsa_circ_0003090*, that can differentiate children with SLE from healthy controls (Li et al., 2019b). Zhao et al. also identified and validated *hsa_circ_0054633* in peripheral whole blood as a sensitive and specific diagnostic biomarker for prediabetes and T2DM (Zhao et al., 2017b). In addition to the above examples, the potential of using blood circRNAs as disease biomarkers has been explored for many other human diseases, such as coronary artery disease (Zhao et al., 2017a; Wang et al., 2019a; Liang et al., 2020), community‐acquired pneumonia (Zhao et al., 2019), and schizophrenia (Yao et al., 2019). A list of current proposed potential blood circRNA biomarkers is listed in Table 2.

**Table 2:** CircRNA biomarkers in blood cells or whole blood.

**Disease**	**CircRNA biomarker**	**Source**	**Expression change**	**Cohort size**	**Clinical significance**	**AUC**	**Method**	**Reference**
AIS	Circ-DLGAP4	PBMC	Down	170 AIS /170 HC	Associated with Health Stroke Scale scor**e** and levels of C-reactive protein, TNF-α, IL-6, IL-8, IL-22.	0.816	RT-qPCR	Zhu et al. (2019b)
ALS	Hsa_circ_0023919 Hsa_circ_0063411 Hsa_circ_0088036	Leukocyte	Down Up Up	60 ALS/15 HC	Hsa_circ_0063411 was associated with the disease duration and survival time.	0.950	Microarray RT-qPCR	Dolinar et al. (2019)
AML	Hsa_circ_0004277	Mononuclear cell	Down	115 AML/12 HC	Associated with progressive stage.	0.957	Microarray RT-qPCR	Li et al. (2017c)
CAD	CircZNF609	Leukocyte	Down	330 CAD/209 HC	Associated with inflammatory processes.	0.761	RT-qPCR	Liang et al. (2020)
CAD	Hsa_circ_0001879 Hsa_circ_0004104	PBM	Up	436 CAD/297 HC	Hsa_circ_0001879 was associated with body mass index (BMI), systolic blood pressure, diastolic blood pressure and Gensini score; hsa_circ_0004104 was associated with high-density lipoprotein cholesterol.	0.703 0.700	Microarray RT-qPCR	Wang et al. (2019a)
CAD	Hsa_circ_0124644	Whole blood	Up	179 CAD/157 HC	Associated with severity.	0.769	Microarray RT-qPCR	Zhao et al. (2017a)
CAP	Hsa_circ_0018429 Hsa_circ_0026579 Hsa_circ_0099188 Hsa_circ_0012535	Whole blood	Up	36 CAP/36 HC	NA	0.878	Microarray RT-qPCR	Zhao et al. (2019)
CML	Hsa_circ_100053	PBMC	Up	150 CML/100 HC	Associated with clinical stage, BCR/ABL mutant status, imatinib response and prognosis.	NA	RNA-seq RT-qPCR	Ping et al. (2019)
CSCC	Hsa_circ_0101996 Hsa_circ_0101119	Whole blood	Up	87 CSCC/55 HC	NA	0.964	RT-qPCR	Wang et al. (2017a)
EH	Hsa_circ_0037911	Whole blood	Up	100 EH/100 HC	Associated with gender, smoking, drinking and serum creatinine.	0.627	Microarray RT-qPCR	Bao et al. (2018)
EH	Hsa_circ_0037909	Whole blood	Up	48 EH/48 HC	Associated with serum creatinine and low‐density lipoprotein.	0.682	RT-qPCR	Bao et al. (2019)
EH	Hsa_circ_0014243	Whole blood	Up	89 EH/89 HC	Associated with age, high-density lipoprotein level and glucose level.	0.732	RT-qPCR	Zheng et al. (2019)
EH	Hsa_circ_91025	Whole blood	Up	96 EH/96 HC	Associated with high‐density lipoprotein, BMI, diastolic blood pressure and systolic blood pressure.	0.620	RT-qPCR	Zheng et al. (2020)
GC	Hsa_circ_0001821	Whole blood	Down	30 GC/30 HC	NA	0.872	RT-qPCR	Kong et al. (2019)
HCC	Hsa_circ_0000798	PBMC	Up	72 HCC/30 HC	Associated with tumor size, cirrhosis and overall survival.	0.703	RNA-seq RT-qPCR	Lei et al. (2019)
HT	Hsa_circ_0089172	PBMC	Up	35 HT/35 HC	Associated with the serum level of the thyroid peroxidase antibody.	0.715	RNA-seq RT-qPCR	Xiong et al. (2019)
IA	Hsa_circ_0021001	Whole blood	Down	223 IA/131 HC	Associated with aneurysm rupture, Hunt, Hess level, timing of surgery, disease-free survival and overall survival.	0.870	RT-qPCR	Teng et al. (2017)
MI	MICRA	Whole blood	Down	642 Acute MI/86 HC	Associated with the risk of left ventricular dysfunction.	NA	Microarray RT-qPCR	Vausort et al. (2016)
MS	Hsa_circ_0005402 Hsa_circ_0035560	Leucocyte	Down	45 MS/26 HC	NA	0.899 0.706	Microarray RT-qPCR	Iparraguirre et al. (2017)
NSCLC	Hsa_circ_0102533	Whole blood	Up	41 NSCLC/26 HC	Associated with tumor type, TNM stage, LNM and distant metastasis.	0.774	Microarray RT-qPCR	Zhou et al. (2018b)
OA	Hsa_circ_0032131	Whole blood	Up	25 OA/25 HC	Associated with the pathological process.	0.846	Microarray RT-qPCR	Wang et al. (2019f)
KBD / OA	Hsa_circ_0020014	Whole blood	Down	25 KBD/25 OA	Associated with early diagnosis of OA and KBD.	0.642	Microarray RT-qPCR	Wang et al. (2020e)
PE	Hsa_circ_0004904 Hsa_circ_0001855	Whole blood	Up	35 PE/35 HC	Associated with serum pregnancy-associated plasma protein A level.	0.611 0.621	Microarray RT-qPCR	Jiang et al. (2018)
PE	Hsa_circ_101222	Blood corpuscle	Up	41 PE/41 HC	NA	0.706	Microarray RT-qPCR	Zhang et al. (2016)
PMOP	Hsa_circ_0001275	PBMC	Up	58 PMOP/41 HC	Associated with T-score.	0.759	Microarray RT-qPCR	Zhao et al. (2018a)
RA	Hsa_circ_0044235	Whole blood	Down	77 RA/31 SLE/50 HC	NA	0.779	RT-qPCR	Luo et al. (2018)
RA	Hsa_circ_104871 Hsa_circ_003524 Hsa_circ_101873 Hsa_circ_103047	PBMC	Up	35 RA/30 HC	NA	0.833 0.683 0.676 0.671	Microarray RT-qPCR	Ouyang et al. (2017)
RA	Hsa_circ_0000396 Hsa_circ_0130438	PBMC	Down	32 RA/20 HC	NA	0.809 0.774	RNA-seq RT-qPCR	Yang et al. (2019)
SLE	Hsa_circ_0000479	PBMC	Up	97 SLE/50 RA/89 HC	Associated with albumin level, urine protein, IgG, leukocytes, hemoglobin and ESR.	0.731	RNA-seq RT-qPCR	Guo et al. (2019)
SLE	Hsa_circ_0057762 Hsa_circ_0003090	Whole blood	Up	24 SLE/24 HC	Hsa_circ_0057762 was associated with the SLEDAI-2K score.	0.804 0.848	Microarray RT-qPCR	Li et al. (2019b)
SLE	Hsa_circ_0044235 Hsa_circ_0068367	PBMC	Down	79 SLE/30 RA/62 HC	Hsa_circ_0044235 was associated with the numbers of monocytes and autoantibodies.	0.873 0.768	Microarray RT-qPCR	Luo et al. (2019)
SLE	CircPTPN22	PBMC	Down	53 SLE/40 HC	Associated with SLE activity index scores.	0.918	RNA-seq RT-qPCR	Miao et al. (2019)
SLE	Hsa_circ_407176 Hsa_circ_001308	PBMC	Down	126 SLE/102 HC	NA	0.806 0.722	Microarray RT-qPCR	Zhang et al. (2018e)
SLE	Hsa_circ_0012919	CD4^+^ T cell	Up	28 SLE/18 HC	Associated with clinical and lab features.	–	Microarray RT-qPCR	Zhang et al. (2018a)
Schizophrenia	Hsa_circ_104597	PBMC	Down	102 Schizophrenia/103 HC	Significant expression difference between pre-treatment and post-treatment.	0.885	Microarray RT-qPCR	Yao et al. (2019)
T2DM	CircANKRD36	Leucocyte	Up	43 T2DM/45 HC	Associated with glucose, glycosylated hemoglobin and interleukin.	NA	RNA-seq RT-qPCR	Fang et al. (2018)
T2DM	Hsa_circ_0054633	Whole blood	Up	90 T2DM/83 Pre-diabetes/86 HC	NA	0.751	Microarray RT-qPCR	Zhao et al. (2017b)
TB	Hsa_circ_103017 Hsa_circ_059914 Hsa_circ_101128	PBMC	Up	31 TB/30 HC	Hsa_circ_101128 was associated with the level of let‐7a.	0.870 0.821 0.817	Microarray RT-qPCR	Fu et al. (2019)
TB	Hsa_circ_0043497 Hsa_circ_0001204	Monocyte	Up	101 TB/88 HC	Significant expression difference between pre-treatment and post-treatment.	0.860 0.848	Microarray RT-qPCR	Huang et al., (2017c)
TB	Hsa_circ_001937	PBMC	Up	178 TB/40 PE/40 COPD/40 LC/133 HC	Associated with radiological scores; significant expression difference between pre-treatment and post-treatment.	0.873	Microarray RT-qPCR	Huang et al., (2018c)
TB	Hsa_circ_0001380	PBMC	Down	32 TB/31 HC	NA	0.950	RT-qPCR	Luo et al. (2020b)
TB	Hsa_circ_0000414 Hsa_circ_0000681 Hsa_circ_0002113 Hsa_circ_0002362 Hsa_circ_0002908 Hsa_circ_0008797 Hsa_circ_0063179	PBMC	Up	12 TB/13 HC	NA	0.946	RNA-seq Microarray RT-qPCR	Qian et al. (2018)
TB	Hsa_circ_0005836	PBMC	Down	49 TB/45 HC	NA	NA	RNA-seq RT-qPCR	(Zhuang et al., 2017)

**Abbreviation:** AIS: acute ischemic stroke; ALS: amyotrophic lateral sclerosis; AML: acute myeloid leukemia; CAD: coronary artery disease; CAP: community‐acquired pneumonia; CML: chronic myeloid leukemia; COPD: chronic obstructive pulmonary disease; CSCC: cervical squamous cell carcinoma; EH: essential hypertension; GC: gastric cancer; HC: healthy control; HCC: hepatocellular carcinoma; HT: Hashimoto’s thyroiditis; IA: intracranial aneurysm; KBD: Kashin-Beck disease; MI: myocardial infarction; MS: multiple sclerosis; NA: not applicable; NSCLC: non-small cell lung cancer; OA: osteoarthritis; PBMC: peripheral blood mononuclear cell; PE: pre-eclampsia; PMOP: postmenopausal osteoporosis; RA: rheumatoid arthritis; SLE: systemic lupus erythematosus; T2DM: type 2 diabetes mellitus; TB: tuberculosis.

## CONCLUSIONS AND FUTURE PERSPECTIVES

The high stability, abundance and spatiotemporal specific expression of blood circRNAs make them ideal biomarkers for liquid biopsy. In the past several years, many studies have shown that blood circRNAs, both cell-free blood circRNAs and circRNAs in blood cells, have great potential as biomarkers of many human diseases in liquid biopsy. A biomarker with broad clinical application must have demonstrated analytical validity, clinical validity and clinical utility (Byron et al., 2016). Therefore, several issues need to be considered and investigated before peripheral blood circRNA biomarkers can be translated into clinical practice. First, a blood circRNA-based gene test should prove its analytical validity within clinically relevant conditions. Although substantial advances have been made in the past several years (Szabo and Salzman, 2016; Kristensen et al., 2019), the methods to discover and profile circRNAs are far from optimal. Future studies need to test the analytical performance of different circRNA profiling methods in clinical blood samples, such as RNA-seq, circRNA microarray, reverse transcription quantitative PCR (RT-qPCR) and RT-ddPCR (Kristensen et al., 2019). In estimating analytical sensitivity and specificity, reference standards that can be specifically applied in circRNA discovery and profiling are needed (Hardwick et al., 2017). Furthermore, the procedure to discover and validate blood circRNA biomarkers needs to be standardized, including blood collection and preservation, circRNA extraction, library construction, and computational analysis (Byron et al., 2016; Anfossi et al., 2018). With the use of a standardized procedure, future studies need to estimate the technical robustness and reproducibility of the proposed biomarkers within and between laboratories. Second, the blood circRNA biomarkers identified in current studies (Tables 1 and 2) are only preliminary biomarker signatures for human diseases. The designed experiments in most studies are case-control studies of samples with well-defined phenotypes, and the sample size is relatively small. To test their clinical validity, more clinical samples are required to validate their sensitivity and specificity in a larger cohort, especially their performance in discriminating patients with similar clinical phenotypes. Moreover, their performance in diagnosing the disease or predicting the disease outcome also needs to be tested in a prospective cohort in a clinical practice setting. Finally, further studies are needed to test and validate the usefulness of these biomarkers in clinical practice, such as their ability to inform clinical decisions and improve outcomes. Although many challenges and problems need to be solved, the promising potential of translating blood circRNA biomarkers into the clinic brings us new and inspiring options for liquid biopsy.
